# A Cross-Sectional Study on the Association of Walnut Consumption with Obesity and Relative Fat Mass among United States Adolescents and Young Adults in NHANES (2003–2020)

**DOI:** 10.1016/j.cdnut.2024.104407

**Published:** 2024-06-28

**Authors:** Nana Gletsu-Miller, Beate Henschel, Carmen D Tekwe, Krisha Thiagarajah

**Affiliations:** 1 Department of Applied Health Science, School of Public Health, Indiana University, Bloomington, IN, United States; 2 Department of Epidemiology and Biostatistics, School of Public Health, Indiana University, Bloomington, IN, United States

**Keywords:** NHANES, adolescents and young adults, walnuts, nuts, relative fat mass, obesity

## Abstract

**Background:**

Walnuts contain nutrients and phytochemicals that can promote metabolic health. However, the high energy content of walnuts along with other nuts raises the concern that consuming nuts promotes obesity.

**Objectives:**

We sought to investigate the associations between consumption of walnuts as well as other nuts and measures of obesity in adolescents and young adults.

**Methods:**

This study included 8874 adolescents (12–19 y) and 10,323 young adults (20–39 y) from 8 waves of National Health and Nutrition Examination Survey data (2003–2020). The associations of consumption of *1*) walnuts only (WO); *2*) walnuts with other nuts (WON); *3*) other nuts (ON); and *4*) no nuts (NN) with obesity status and relative fat mass (RFM) were assessed using logistic and linear regressions stratified by age group and sex. Sample weights were used in all statistical analyses.

**Results:**

The mean daily intake of walnuts was not different between the 2 walnut consumption groups within each age group (adolescents: 2.18 [standard error (SE) 0.14] g; *P* = 0.917; young adults: 4.23 [0.37] g; *P* = 0.682). The WON group had the lowest prevalence of obesity (adolescents: 8.3%; young adults: 21.1%) while the NN group had the highest prevalence (adolescents: 24.1%; young adults: 35.4%). The models indicated lower odds of obesity in adolescent girls (odds ratio [OR]: 0.27; *P* < 0.05) and young adult women (OR: 0.58; *P* < 0.05) who consumed WON than in those who consumed NN. In both young women and girls, RFM was significantly lower in the WON and ON groups than the NN group (*P* < 0.001). In young men, WON consumption was also associated with a lower RFM (OR: −1.24; 95% confidence interval: −2.21, −0.28) compared with NN consumption.

**Conclusions:**

For adolescents girls and young women, dietary intake of walnuts combined with other nuts has the strongest inverse association with measures of obesity.

## Introduction

The prevalence of obesity in the United States increased from 14.8% in 1999–2000 to 21.2% in 2017–2018 among adolescents [1] and from 26.1% to 40.0% among young adults [2]. Obesity is considered a major public health concern because it is a risk factor for noncommunicable chronic diseases [3]. Nonetheless, the long-term consequences of being overweight or obese and the difficulty in losing weight and maintaining weight loss underscore the importance of obesity prevention.

The 2020–2025 Dietary Guidelines for Americans (DGA) reports that the poor dietary quality of the American diet manifests as nutrients that are underconsumed relative to recommendations. Calcium, potassium, magnesium, dietary fiber, and vitamin D are considered dietary components of public health concern for the general United States population [4]. Shifts in eating patterns to increase consumption of vegetables, fruits, whole grains, and nuts and seeds along with dairy can help consumers move closer to meeting nutrient recommendations to prevent chronic diseases. Nuts are considered a core element of healthy dietary patterns such as the healthy Mediterranean, vegetarian, and United States style patterns [4]. Nuts are a nutrient-dense food group, and they provide a range of nutrients, including fiber, magnesium, and potassium, that are often lacking in the typical United States diet [4].

A previous study from our group, using modeling analysis of dietary data from the United States NHANES, indicated that adding walnuts to the usual diet of children, adolescents, and adults can improve diet quality [5]. However, nuts are calorie dense, leading to the concern that including nuts in the diet could promote obesity. Despite nuts being calorie dense, consumption of nuts has been associated with body weight management [6,7]; therefore, nuts may be included in a healthy eating pattern to prevent or manage obesity [8,9]. The high fat content of nuts increases energy intake; however, the calories in nuts come from unsaturated fatty acids (both MUFAs and PUFAs). These fats are considered healthy [10] and encouraged by the DGA [4]. Compared to other nuts, walnuts are particularly rich in α-linolenic acid (ALA), an ω-3 PUFA [11]. The high content of ALA in walnuts is notable because ALA may play an important role in managing obesity and related diseases [[12], [13], [14]].

The existing literature on associations between nut consumption and obesity focuses on older adults [15,16], leaving a gap in research on nut consumption and the risk of obesity in adolescents or young adults. In the present study, using nationally representative United States data from NHANES, we sought to investigate the association between nut consumption and relative fat mass (RFM) and obesity among adolescents and young adults. To explore whether associations among measures of obesity and walnut consumption were distinct from those of other nuts, we subdivided the categories of nut consumption into walnuts only (WO), walnuts with other nuts (WON), other nuts (ON), and no nuts (NN). We hypothesized that compared to no consumption of nuts, consumption of nuts, especially walnuts, is associated with a lower prevalence of obesity and lower RFM.

## Methods

### NHANES study population

The NHANES dataset comes from a nationally representative sample of the United States noninstitutionalized civilian population [17]. NHANES data are collected by the National Center for Health Statistics (NCHS) of the Centers for Disease Control and Prevention. Written informed consent was obtained from the participants or their proxies, and the survey protocol was approved by the Research Ethics Review Board at the NCHS. For the current study, we used data from 8 NHANES cycles: 2003–2004, 2005–2006, 2007–2008, 2009–2010, 2011–2012, 2013–2014, 2015–2016, and 2017–March 2020. The focus of this study was on adolescents (ages 12–19 y) and young adults (20–39 y). NHANES generally defines the age of adolescence starting at age 12 y [18]. Children <12 y and adults ≥40 y were excluded. Further, those who reported implausible energy intakes (<500 kcal or >5000 kcal/day; *n* = 314) were excluded.

### Measures of BMI and RFM

Body measurement data, including height, weight, and waist circumference, were obtained by trained medical workers during physical examinations following standard procedures [17]. BMI was calculated by dividing weight (in kilograms) by the square of height (in meters) to determine the degree of obesity [19]. For young adults, BMI ≥30 kg/m^2^ was considered obese [20]. For adolescents, we calculated BMI-for-age percentiles, and obesity was defined as BMI-for-age percentile ≥95th percentile [21].

Because the accuracy of BMI as a measure of body fatness has been debated, we calculated RFM, which has been validated against total body fat and regional fat composition [22]. For young adults and older adolescents (15–19 y), we calculated RFM using the following equation:

(1) $$RFM=\left\{ \begin{array}{c} 64-20*\frac{height}{waist\;circumference},men\\ 76-20*\frac{height}{waist\;circumference},women \end{array}\right.$$

where both height and waist circumference are expressed in meters. For younger adolescents (12–14 y), we used the pediatric equation for RFM (RFMp) [23]:

(2) $${RFM}_{p}=\left\{ \begin{array}{c} 74-22*\frac{height}{waist\;circumference},boys\\ 79-22*\frac{height}{waist\;circumference},girls \end{array}\right.$$

where both height and waist circumference are expressed in meters.

### Dietary intake interview

Dietary intake interviews were conducted in-person in the NHANES mobile examination clinics by trained dietary interviewers [24]. The specific types and amounts of food consumed the previous day were recalled by survey participants with the help of recall cue items such as a food model booklet, measuring cups/spoons, bowls, and rulers. A second dietary recall was collected by telephone in all participants 3 to 10 days later using the same recall cues as in the first recall. Similar to the process described by Arab et al. [25], we classified walnut consumption using data from both days of dietary recall such that participants reporting walnut consumption in either recall were classified as walnut consumers.

To identify foods that described or included walnuts, we searched for the word walnuts in the food code and ingredient label descriptors in the year-specific ingredient database, Food and Nutrient Database for Dietary Studies [26]. To identify foods that described or included other nuts, we searched for the terms “nut,” “mixed nut,” “almond,” “brazil,” “cashew,” “hazelnut,” “macadamia,” “peanut,” “pecan,” “pine nut,” and “pistachio.” We did not count any nut-based milk, nut oils, coconut, chestnut, butternut, nutmeg, and soy nut as nut items. For all walnut-containing food items, we obtained the proportional weight of walnuts of the total food item weight.

#### Walnut food classification

Food codes were categorized into the following categories: WO; WON, which included peanuts; peanuts but no other nuts; ON; and NN [25]. We separated out peanut-only foods because peanuts are not tree nuts.

#### Walnut consumption groups

The walnut food code classification was merged with the participants’ dietary recall information in the next step. This allowed the categorization of participants into 4 different groups. Participants that reported consumption of foods that contained walnuts, but no other nuts, were coded as WO consumers. Likewise, participants that reported consuming both walnut foods and other nut containing foods, were classified as WON consumers. Participants that reported eating foods containing tree nuts that are not walnuts were classified as ON consumers. Participants whose only nut consumption was peanuts, and no other nuts, were excluded from the analysis [25]. All other participants (i.e., those that did not report consuming any walnuts or other nuts) were classified as NN consumers.

In addition to the categorical measure of nut consumption, we calculated the amounts of walnuts consumed across all walnut-containing foods consumed on that day as the total per individual. The amount assigned to walnut intake for mixed foods was calculated using the proportional weight of walnuts times the total food item weight.

### Demographic factors and covariates

We were guided by the Accumulating Data to Optimally Predict Obesity Treatment conceptual framework, which illustrates the relationship between obesity and risk factors [27], and we included covariates within behavior, biological, and psychosocial domains. Specifically, covariates were physical activity, smoking status, as well as energy intake, diet quality, and alcohol intake representing the behavior domain; age and race/ethnicity within the biological domain; and socioeconomic status for the psychosocial domain. Race/ethnicity was categorized into Mexican American or Hispanic, non-Hispanic White, non-Hispanic Black, and other races. Smoking status was self-reported smoking in the past 5 d and categorized into nonsmoker and current smoker [28]. Physical activity status was categorized, using the time spent in moderate and vigorous recreational activities, as inactive (0 min/wk), insufficiently active (1–149 min/wk), sufficiently active (150–299 min/wk), and highly active (≥300 min/wk) [29]. For young adults only, alcohol intake was dichotomized using past year alcohol consumption (≥12 drinks in the past year) into drinkers and nondrinkers [30]. The 2015 Healthy Eating Index (HEI) was used as a composite measure of diet quality [31]. For easier interpretation of intercept and coefficients, energy intake, HEI-2015 diet quality scores, and age were mean centered within age and sex groups.

### Statistical analyses

This analysis utilized sampling strata, clusters, and survey weights in line with the NHANES analytical guidelines, ensuring the results could be generalized to the United States population. Two-year sample weights for each NHANES cycle were combined to provide 16-y weights for the 2003–2020 survey period [32].

Descriptive statistics are presented as mean ± SE for continuous data and proportions for categorical data. Differences between nut consumption groups were calculated using the Rao–Scott chi-square goodness-of-fit test for categorical variables and bivariate linear regression for continuous variables. To analyze the associations of nut consumption categories with obesity and RFM, we used regression models adjusted for age, race/ethnicity, alcohol intake in the past year (young adults only), smoking status, energy intake, diet quality using HEI-2015, socioeconomic status using the income to poverty ratio, and physical activity. We present odds ratios (ORs) with 95% confidence intervals (CIs) from logistic regression models for the binary obesity outcome, while β coefficients with 95% CIs are shown as results from linear regression models for the continuous outcome of RFM. All analyses were stratified by age group (adolescents compared with young adults) and sex (male compared with female). We used SAS software (version 9.4, SAS Institute Inc.) for all data analyses with an α level of 0.05.

## Results

### Description of the sample

**FIGURE 1:**
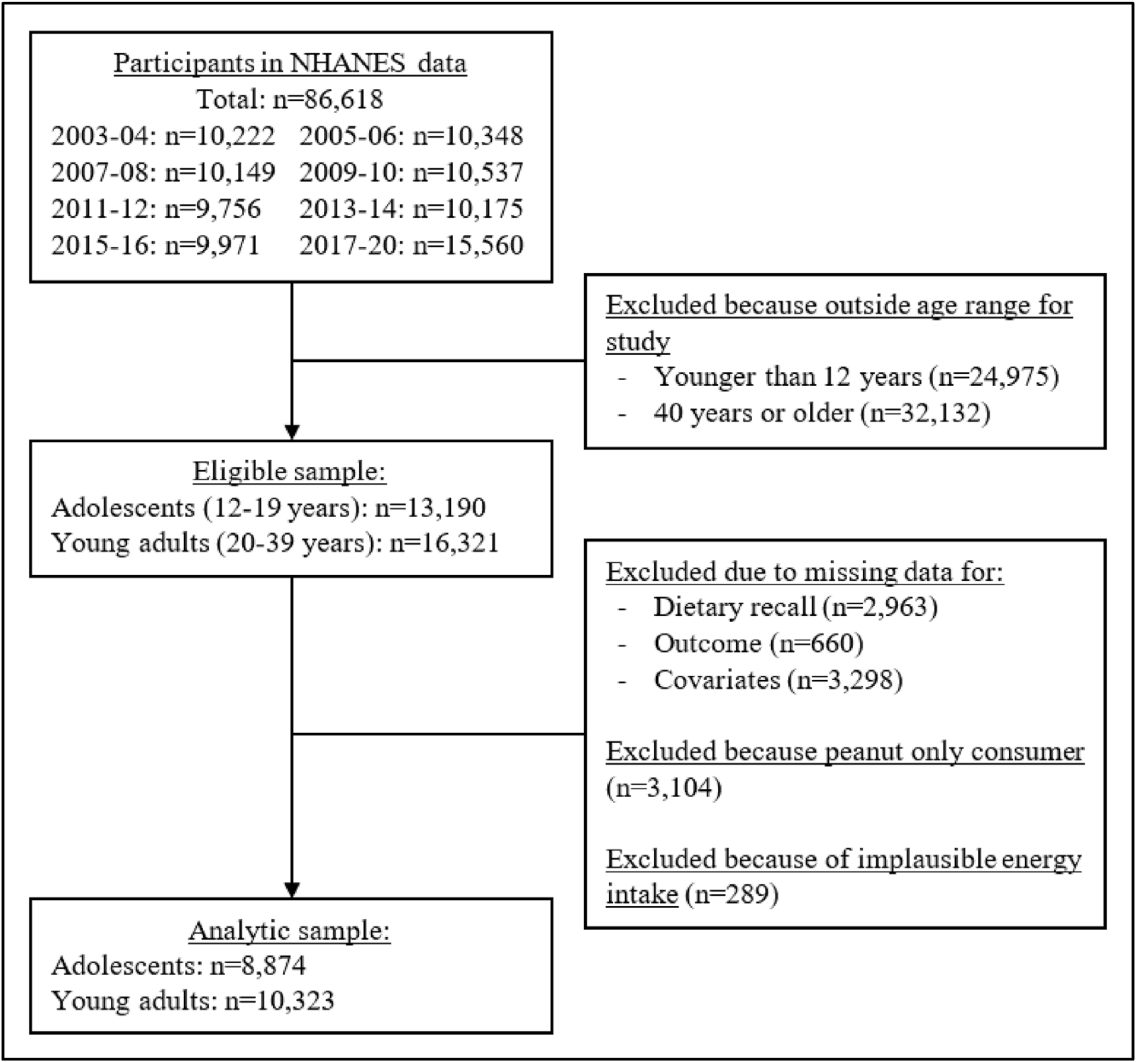
Flow chart of study selection for adolescents and young adults. NHANES, National Health and Nutrition Examination Survey.

The sample included 8874 adolescents and 10,323 young adults, as summarized in Figure 1. Presented in Table 1 are the characteristics of participants who were included in our analysis. In the adolescent population, 3.8%, 3.8%, 16.1%, and 76.3% reported consuming WO, WON, ON, and NN, respectively. Among the young adult population, 3.8%, 5.5%, 21.6%, and 69.0% reported consuming WO, WON, ON, and NN, respectively. There were higher proportions of females, non-Hispanic Whites, nonsmokers, higher household income to poverty ratio, and highly active participants in the WON group than the other groups (*P* ≤ 0.01). Further, energy intake in the adolescent population was 2008 ± 18, 2110 ± 41, 2202 ± 92, and 2297 ± 64 kcals/d among the NN, ON, WO, and WON groups, respectively (*P* < 0.001 between groups). A similar pattern of energy intake was observed among young adults (2180 ± 14, 2279 ± 22, 2370 ± 57, and 2416 ± 62 kcal/d for the NN, ON, WO, and WON groups, respectively, *P* < 0.001). The average walnut consumption (among walnut consumers) was 2.18 ± 0.14 g/d among adolescents and 4.23 ± 0.37 g/d among young adults (Table 2), with no significant differences between WO and WON consumers (*P* = 0.917 for adolescents, *P* = 0.682 for young adults).

**TABLE 1:** Demographic characteristics (*n* = 8874 adolescents; *n* = 10,323 young adults).

	Adolescents					Young adults				
	Walnut classification				Total or *P*	Walnut classification				Total or *P*
	Walnuts only	Walnuts with other nuts	Other nuts	No nuts		Walnuts only	Walnuts with other nuts	Other nuts	No nuts	
Overall	314 (3.8%)	236 (3.8%)	1195 (16.1%)	7129 (76.3%)	8874	374 (3.8%)	442 (5.5%)	2007 (21.6%)	7500 (69%)	10,323
Age, y					0.472					<0.001
	15.6 (0.2)	15.2 (0.2)	15.5 (0.1)	15.5 (<0.1)	15.5 (<0.1)	29 (0.4)	31.4 (0.4)	30 (0.2)	29 (0.1)	29.3 (0.1)
Sex					<0.001					<0.001
Female	172 (52.9%)	140 (62.3%)	663 (58.0%)	3390 (47.6%)	4365	210 (52.8%)	262 (58.5%)	1185 (54.2%)	3739 (48.0%)	5396
Male	142 (47.1%)	96 (37.7%)	532 (42.0%)	3739 (52.4%)	4509	164 (47.2%)	180 (41.5%)	822 (45.8%)	3761 (52.0%)	4927
Race/ethnicity					<0.001					<0.001
Mexican American or Hispanic	86 (15.7%)	54 (10.5%)	415 (18.4%)	2408 (21.9%)	2963	83 (12.2%)	66 (10.1%)	522 (16.9%)	2202 (21.5%)	2873
Non-Hispanic Black	66 (10.4%)	32 (4.6%)	203 (8.4%)	2223 (18.5%)	2524	57 (9.4%)	48 (5.0%)	282 (7.5%)	1803 (15.8%)	2190
Non-Hispanic White	129 (65.9%)	118 (77.4%)	403 (63.5%)	1831 (51.6%)	2481	188 (72.3%)	262 (75.9%)	908 (65.8%)	2687 (54.4%)	4045
Other race	33 (8.0%)	32 (7.5%)	174 (9.7%)	667 (8.0%)	906	46 (6.1%)	66 (9.0%)	295 (9.8%)	808 (8.3%)	1215
Household income to poverty ratio					<0.001					<0.001
	3.0 (0.2)	3.2 (0.2)	2.9 (0.1)	2.3 (0.1)	2.5 (0.1)	3.2 (0.1)	3.6 (0.1)	3 (0.1)	2.5 (0)	2.7 (0)
Smoking					0.001					<0.001
Current smoker	32 (12.5%)	13 (4.5%)	98 (9.6%)	788 (14.1%)	931	103 (30%)	71 (16.8%)	439 (21.9%)	2616 (36.4%)	3229
Nonsmoker	282 (87.5%)	223 (95.5%)	1097 (90.4%)	6341 (85.9%)	7943	271 (70%)	371 (83.2%)	1568 (78.1%)	4884 (63.6%)	7094
Alcohol drinking										0.547
Drinker in past year	—	—	—	—	—	274 (80%)	337 (80%)	1510 (80.7%)	5472 (78.3%)	7593
Nondrinker	—	—	—	—	—	100 (20%)	105 (20%)	497 (19.3%)	2028 (21.7%)	2730
Energy intake, kcal					<0.001					<0.001
	2202 (92)	2297 (64)	2110 (41)	2008 (18)	2043 (15)	2370 (57)	2416 (62)	2279 (22)	2180 (14)	2222 (12)
HEI-2015 score					<0.001					<0.001
	45.1 (0.8)	49.6 (1.3)	52.3 (0.6)	44.5 (0.2)	46.0 (0.2)	50.5 (0.9)	58.9 (0.9)	57.1 (0.5)	47.0 (0.2)	50.0 (0.3)
Recreational physical activity					0.008					<0.001
Inactive	49 (12%)	33 (14.5%)	225 (15.4%)	1484 (20.3%)	1791	133 (30.0%)	103 (24.5%)	648 (27.3%)	3410 (41.3%)	4,294
Insufficiently active	67 (18.6%)	28 (8.8%)	158 (11.3%)	1114 (14.7%)	1367	78 (18.4%)	85 (17.7%)	366 (18%)	1190 (17.6%)	1719
Sufficiently active	40 (16.8%)	39 (16.0%)	179 (14.2%)	1023 (12.9%)	1281	51 (16.4%)	67 (13.7%)	275 (14.1%)	849 (12.4%)	1242
Highly active	158 (52.6%)	136 (60.7%)	633 (59.2%)	3508 (52.1%)	4435	112 (35.2%)	187 (44.1%)	718 (40.7%)	2051 (28.7%)	3068

Abbreviation: HEI, Healthy Eating Index.

Data shown as weighted means (weighted SE) for continuous variables, and unweighted counts [weighted proportions (column percentage)] for categorical variables. *P* values for differences between walnut groups within age group were obtained by weighted linear regression models for age and calories, and Rao–Scott chi-square test for all other variables.

**TABLE 2:** Grams of walnuts consumed, obesity prevalence, and relative fat mass by walnut consumption category, age group, and sex (*n* = 8874 adolescents; *n* = 10,323 young adults).

Age group	Overall	Walnut classification				*P* for group differences
		Walnut only	Walnut with other nuts	Other nuts	No nuts	
Adolescents (12-19 y)						
Walnut consumption, g	2.18 (0.14)	2.2 (0.23)	2.16 (0.23)	0 (0)	0 (0)	0.917
Girls	1.87 (0.15)	1.63 (0.20)	2.08 (0.23)	0 (0)	0 (0)	0.148
Boys	2.60 (0.26)	2.84 (0.37)	2.30 (0.50)	0 (0)	0 (0)	0.445
Obesity prevalence	21.9%	20.4%	8.3%	15.1%	24.1%	<0.001
Girls	21.3%	23.1%	6.6%	12.4%	24.5%	<0.001
Boys	22.5%	17.3%	11.3%	19.0%	23.7%	0.053
RFM	29.6 (0.2)	29.3 (0.6)	28.6 (0.8)	29.5 (0.3)	29.7 (0.2)	0.574
Girls	35.3 (0.2)	34.3 (0.7)	32.5 (0.6)	34 (0.3)	35.8 (0.2)	<0.001
Boys	23.9 (0.2)	23.7 (0.7)	22.2 (0.9)	23.2 (0.3)	24.1 (0.2)	0.046
Young adults (20–39 y)						
Walnut consumption, g	4.23 (0.37)	4.06 (0.43)	4.35 (0.56)	0 (0)	0 (0)	0.682
Women	3.96 (0.45)	3.90 (0.56)	4.01 (0.64)	0 (0)	0 (0)	0.899
Men	4.57 (0.60)	4.23 (0.68)	4.84 (0.91)	0 (0)	0 (0)	0.583
Obesity prevalence	32.6%	25.4%	21.1%	28.2%	35.4%	<0.001
Women	33.7%	22.5%	19.6%	27.8%	37.8%	<0.001
Men	31.6%	28.6%	23.2%	28.7%	33.1%	0.095
RFM	33.3 (0.2)	32.9 (0.6)	32.7 (0.5)	33.1 (0.3)	33.4 (0.2)	0.319
Women	39.9 (0.2)	38.6 (0.6)	37.8 (0.5)	38.8 (0.3)	40.6 (0.2)	<0.001
Men	26.7 (0.1)	26.5 (0.5)	25.5 (0.5)	26.2 (0.3)	26.9 (0.2)	0.017

Abbreviations: BMI, body mass index; RFM, relative fat mass; SE, standard error.

Data shrhown as weighted means (weighted SE) for walnut consumption and RFM, and weighted proportions (percentage) for obesity prevalence. Obesity prevalence: adolescents: BMI-for-age percentile ≥95th percentile; young adults: BMI ≥30 kg/m^2^. RFM for adolescents aged 12–14 y was calculated using the pediatric RFM equation: RFM = 74−(22 × height/waist circumference) + 5 × sex, where sex = 1 for girls and sex = 0 for boys and height and waist circumference are expressed in meters. RFM for adolescents aged 15–19 y and young adults was calculated using the following RFM equation: RFM = 64−(20 × height/waist circumference) +12 × sex, where sex = 1 for women and sex = 0 for men and height and waist circumference are expressed in meters. *P* values for differences between walnut groups within age group were obtained by weighted linear regression models for walnut consumption and RFM, and Rao–Scott chi-square test for obesity prevalence. For walnut consumption, overall value and *P* value only used the 2 groups with walnut consumption.

#### Associations of walnut consumption with obesity

In both adolescents and young adults, the prevalence of obesity was lowest in the WON group compared with the other groups (*P* < 0.001, Table 2). In multivariable adjusted logistic regression models, the odds of having obesity were significantly lower in WON consumers, compared with NN consumers, with all other covariates being held constant, for adolescent girls and young women (adolescent girls: odds ratio [OR]: 0.27; 95% CI: 0.12, 0.58; *P* < 0.001; young women: OR: 0.58; 95% CI: 0.36, 0.91; *P* = 0.019, Table 3). Additionally, for adolescent girls, compared with NN consumers, the odds of obesity were significantly lower for ON consumers (adolescent girls: OR: 0.50; 95% CI: 0.34, 0.73; *P* < 0.001). Finally, among young women WO consumers, compared with NN consumers, we found significantly lower odds of obesity (OR: 0.60; 95% CI: 0.37, 0.95; *P* = 0.029).

**TABLE 3:** Results from multivariable adjusted logistic regression models for the association of obesity^1^ with walnut consumption and other covariates (*n* = 8874 adolescents; *n* = 10,323 young adults).

Effect	Adolescents		Young adults	
	Boys	Girls	Men	Women
Age^2^, y	0.98 (0.75, 1.28)	0.89 (0.64, 1.23)	1.25 (1.15, 1.35)∗	1.21 (1.12, 1.30)∗
Mexican American or Hispanic	1.34 (1.01, 1.77)∗	0.95 (0.68, 1.33)	1.41 (1.13, 1.75)∗	1.48 (1.19, 1.84)∗
Non-Hispanic Black	1.00 (0.77, 1.31)	1.37 (0.97, 1.94)	1.26 (1.00, 1.58)∗	2.16 (1.75, 2.66)∗
Non-Hispanic White	Ref	Ref	Ref	Ref
Other Race	1.08 (0.67, 1.75)	0.57 (0.37, 0.90)∗	0.76 (0.55, 1.04)	0.69 (0.52, 0.91)∗
Income to poverty ratio	0.96 (0.88, 1.03)	0.86 (0.77, 0.95)∗	1.05 (0.98, 1.12)	0.90 (0.85, 0.96)∗
Current smoker: no	Ref	Ref	Ref	Ref
Current smoker: yes	0.99 (0.69, 1.44)	0.81 (0.52, 1.25)	0.83 (0.68, 1.01)	0.90 (0.71, 1.14)
Drinker in past year: no	—	—	Ref	Ref
Drinker in past year: yes	—	—	0.97 (0.77, 1.23)	0.71 (0.58, 0.88)∗
Energy intake^3^	0.85 (0.79, 0.92)∗	0.90 (0.81, 1.00)∗	0.93 (0.88, 0.98)∗	1.08 (1.01, 1.14)∗
HEI-2015 total score^4^	1.07 (0.95, 1.20)	1.02 (0.91, 1.16)	0.88 (0.81, 0.95)∗	0.87 (0.81, 0.93)∗
Inactive	Ref	Ref	Ref	Ref
Insufficiently active	0.99 (0.61, 1.61)	0.95 (0.65, 1.38)	0.80 (0.60, 1.08)	0.97 (0.79, 1.20)
Sufficiently active	0.71 (0.47, 1.06)	1.09 (0.76, 1.56)	1.07 (0.80, 1.42)	0.85 (0.64, 1.13)
Highly active	0.65 (0.47, 0.90)∗	0.65 (0.48, 0.87)∗	0.68 (0.54, 0.87)∗	0.69 (0.55, 0.87)∗
No nut consumer	Ref	Ref	Ref	Ref
Other nuts consumer	0.82 (0.59, 1.15)	0.50 (0.34, 0.73)∗	0.90 (0.68, 1.20)	0.84 (0.67, 1.05)
Walnut with other nuts consumer	0.52 (0.25, 1.09)	0.27 (0.12, 0.58)∗	0.67 (0.41, 1.09)	0.58 (0.36, 0.91)∗
Walnut only consumer	0.77 (0.40, 1.47)	1.12 (0.57, 2.20)	0.87 (0.53, 1.44)	0.60 (0.37, 0.95)∗

Abbreviations: BMI, body mass index; HEI, Healthy Eating Index; Ref, reference.

Data are shown as odds ratio (95% confidence interval.

1 Obesity defined as BMI-for-age percentile ≥95th percentile (adolescents) or BMI ≥30 kg/m^2^ (young adults).

2 Age was centered at sex and age group specific mean values, per 5 y increase.

3 Energy intake was centered at sex and age group specific mean values, per 500 kcal increase.

4 HEI-2015 total diet quality scores were centered at sex and age group specific mean values, per 10 point increase.

∗ *P* < 0.05.

#### Associations of walnut consumption with RFM

On average, RFM was 29.6 for adolescents and 33.3 for young adults (Table 2). Table 4 shows the associations among nut consumption and RFM (stratified by age group and sex) from multivariable adjusted linear regression models. Among adolescent girls, we also found significantly lower RFM in the WON (β: −2.26; 95% CI: −3.52, −1.00; *P* < 0.001) and ON (β: −1.37; 95% CI: −2.18, −0.56; *P* = 0.001) groups than the NN group. Among young women, we found significantly lower RFM in WON (β: −1.66; 95% CI: −2.65, −0.66; *P* = 0.008) and ON (β: −0.87; 95% CI: −1.48, −0.26; *P* = 0.035) consumers than NN consumers. Among young men, RFM was significantly lower in the WON group (β: −1.24; 95% CI: −2.21, −0.28; *P* = 0.012) than the NN group; however, WO consumption was not significantly different from NN consumption (β: −0.01; 95% CI: −0.87, 0.86; *P* = 0.988). Additionally, WO consumers in all 4 age–sex strata were not significantly different from NN consumers, *P* > 0.05.

**TABLE 4:** Results from multivariable adjusted linear regression models for the associations of relative fat mass with walnut consumption and other covariates (*n* = 8874 adolescents; *n* = 10,323 young adults).

Effect	Adolescents		Young Adults	
	Boys	Girls	Men	Women
Intercept^1^	26.1 (25.0, 27.1)∗∗∗	37.5 (36.3, 38.7)∗∗∗	27.3 (26.5, 28.1)∗∗∗	42.2 (41.4, 42.9)∗∗∗
Age^2^, y	−3.74 (−4.53, −2.96)∗∗∗	2.90 (2.14, 3.67)∗∗∗	1.34 (1.15, 1.53)∗∗∗	0.87 (0.64, 1.10)∗∗∗
Mexican American or Hispanic	0.98 (0.25, 1.70)∗∗	0.63 (−0.21, 1.48)	1.68 (1.09, 2.27)∗∗∗	2.15 (1.53, 2.77)∗∗∗
Non-Hispanic Black	−2.17 (−2.91, −1.43)∗∗∗	−0.43 (−1.47, 0.61)	−1.17 (−1.84, −0.50)∗∗∗	1.54 (0.89, 2.19)∗∗∗
Non-Hispanic White	Ref	Ref	Ref	Ref
Other race	−0.72 (−1.88, 0.43)	−1.61 (−2.49, −0.74)∗∗∗	−0.84 (−1.61, −0.07)∗	−0.82 (−1.55, −0.10)∗
Income to poverty ratio	−0.35 (−0.55, −0.16)∗∗∗	−0.56 (−0.81, −0.31)∗∗∗	0.03 (−0.13, 0.18)	−0.39 (−0.56, −0.22)∗∗∗
Current smoker: no	Ref	Ref	Ref	Ref
Current smoker: yes	0.33 (−0.68, 1.34)	−0.24 (−1.44, 0.96)	−0.86 (−1.31, −0.41)∗∗∗	−0.10 (−0.69, 0.48)
Drinker past year: no	—	—	Ref	Ref
Drinker past year: yes	—	—	0.36 (−0.22, 0.94)	−1.17 (−1.78, −0.55)∗∗∗
Energy intake^3^	−0.53 (−0.73, −0.34)∗∗∗	−0.50 (−0.71, −0.28)∗∗∗	−0.34 (−0.47, −0.22)∗∗∗	0.10 (−0.09, 0.28)
HEI-2015 total score^4^	0.11 (−0.19, 0.42)	0.03 (−0.25, 0.31)	−0.36 (−0.54, −0.19)∗∗∗	−0.29 (−0.47, −0.11)∗∗
Inactive	Ref	Ref	Ref	Ref
Insufficiently active	0.47 (−0.94, 1.87)	0.21 (−0.83, 1.25)	−0.46 (−1.17, 0.26)	−0.25 (−0.85, 0.35)
Sufficiently active	−0.98 (−2.09, 0.12)	0.05 (−0.98, 1.07)	0.16 (−0.62, 0.94)	−0.99 (−1.74, −0.24)∗∗
Highly active	−1.25 (−2.23, −0.26)∗	−0.90 (−1.73, −0.06)∗	−1.31 (−1.81, −0.81)∗∗∗	−1.48 (−2.10, −0.86)∗∗∗
No nuts consumer	Ref	Ref	Ref	Ref
Other nuts consumer	−0.74 (−1.52, 0.03)	−1.37 (−2.18, −0.56)∗∗	−0.49 (−1.12, 0.14)	−0.87 (−1.48, −0.26)∗∗
Walnut with other nuts consumer	−1.40 (−2.94, 0.13)	−2.26 (−3.52, −1.00)∗∗∗	−1.24 (−2.21, −0.28)∗	−1.66 (−2.65, −0.66)∗∗
Walnut only consumer	−0.04 (−1.26, 1.17)	−0.92 (−2.22, 0.37)	−0.01 (−0.87, 0.86)	−1.08 (−2.20, 0.05)

Abbreviations: HEI, Healthy Eating Index; Ref, reference; RFM, relative fat mass.

Data shown as β coefficient (95% confidence interval).

∗∗∗*P* < 0.001; ∗∗*P* < 0.01; ∗*P* < 0.05.

1 Iintercept represents the RFM for an individual with other covariates at reference/mean values.

2 Age was centered at sex and age group specific mean values, per 5 year increase.

3 Energy intake was centered at sex and age group specific mean values, per 500 kcal increase.

4 HEI-2015 total diet quality scores were centered at sex and age group specific mean values, per 10-point increase.

## Discussion

In this study, which used a nationally representative sample of United States adolescents and young adults, the prevalence of obesity was lowest among consumers of WON compared with consumers of WO, ON, and NN. Surprisingly, in both adolescents and young adults, the lowest energy intake was among NN consumers despite the finding of the highest prevalence of obesity in that group, and the highest energy intake was found among WON consumers. Consistent with our finding of the lowest odds of obesity in the WON (compared with NN) group, RFM was lowest among WON compared with NN consumers in both adolescent girls and young adults. Overall, consumption of walnuts in the sample of adolescents and young adults was low in this study. The average consumption of walnuts among adolescents was 2.18 ± 0.14 g while among young adults it was 4.23 ± 0.37 g. This is far below the dietary intake recommendation for nuts of 1 to 1.5 ounces (28.4–42.5 g) per day [4,33].

Our findings also build on our previous study using modeling analysis of dietary data from NHANES, which indicated that adding walnuts to the usual diet of children, adolescents, and adults can improve diet quality [5]. Studies in adults show that consumption of a high-quality diet is inversely associated with BMI; therefore, even though walnuts and other nuts are energy dense, they may not promote obesity [34,35]. A systematic review of cohort studies indicated that long-term moderate intake of nuts (i.e., 1–2 servings of nuts per week) was associated with less weight gain and reduced risk of overweight/obesity [36]. Another meta-analysis of clinical trials indicated that compared to control diets, diets with nuts did not increase body weight or BMI [7]. Indeed, we found in the present study that adolescent and young adult consumers of nuts had higher energy intake than NN consumers but lower prevalence of obesity. Consistent with our study, a study using NHANES data showed that an increase in nuts and seeds consumption led to an increase in caloric intake [37]. Also consistent is a systematic review and meta-analysis of randomized clinical trials [38] that reported that nut consumption was associated with increased energy intake. Another systematic review and meta-analysis of randomized trials reported consumption of nuts or nut products did not lead to weight gain [39]. Our finding is consistent with findings from a similar study by Yang et al. [40] that also indicated that nuts and seed consumption reduced the odds of overweight or obesity among adolescent girls. Because nuts, especially walnuts, are rich in unsaturated fatty acids, one plausible explanation may be the preferential oxidation of long-chain unsaturated fatty acids compared to saturated fatty acids [41,42]. The unsaturated fatty acid ALA, which is high in walnuts compared to other nuts, has been shown to have the highest rate of β oxidation compared to other unsaturated fatty acids [43,44]. Despite high energy content, nut consumption is associated with high thermogenic effects [45], and evidence suggests that MUFAs and PUFAs are more readily oxidized [46] than saturated fat, leading to less body fat accumulation. Furthermore, Atwater factors overestimate the metabolizable energy value of walnuts [47] and other nuts [48,49], and this could explain why consumers of nuts are not susceptible to obesity. In addition, nuts are rich in unsaturated fatty acids, fiber, and protein, which make them a highly satiating food. Therefore, hunger may be suppressed, and subsequently, food intake may be reduced [42]. The satiety effect of nuts may be further enhanced by their physical structure, which requires mastication, thereby activating mechanical, nutrient, and sensory signaling systems that may modify appetitive sensations [50]. The physical structure of nuts leads to fat being contained within cell walls and incompletely digested in the gut resulting in excretion of fat [51], and the poor bioavailability of energy from nuts may be compounded by incomplete mastication [52].

We consistently observed inverse associations among consumption of walnuts with other nuts compared with no nut consumption and obesity in girls and young women, but not in boys or young men. Additionally, young women WO consumers showed significant inverse associations for obesity compared to NN consumers, but we did not find these inverse associations among young men, adolescent boys, or adolescent girls WO consumers. We also observed that girls and young women ON and WON groups showed an inverse association with RFM. Further, among boys or young males, only young males in the WON group showed an inverse association with RFM compared to NN consumers. Burdge et al. [53,54] reported that there is a gender difference in ALA metabolism and storage between young men and women. This may explain the sex differences in associations between nut consumption and lower RFM in the current study.

The strength of this study is that it is one of the few using a large and nationally representative sample of adolescents and young adults. The limitations of the study are that this is an observational study, and the findings cannot infer cause and effect. The fact that we observed less obesity in nut consumers may be due to revere causality, such that people with obesity have altered their diets in terms of nut consumption to prevent more weight gain.

Obesity is the result of complex interactions of multiple factors [27]. To reduce confounding, we controlled for energy intake, diet quality, socioeconomic status, smoking, alcohol intake, physical activity, age, and race/ethnicity and stratified by sex and age group. Consumption of nuts as a behavior seems to be colinear with behaviors and psychosocial factors that are associated with obesity. In both adolescents and young adults, the WON group had a higher income to poverty ratio and were mostly non-Hispanic White females compared to the group that did not consume nuts. Further, WON consumers had a lower prevalence of current smoking and higher physical activity than the group that did not consume nuts. Other studies have shown that poor diet quality and low physical activity are positively associated with obesity [55]. Another trend analysis of obesity using NHANES data revealed that the prevalence of obesity was higher among those who reported lower levels of daily total energy intake, economic status, and physical activity [56] compared to those who reported higher levels.

Future studies should control additional factors such as sleep and other behavioral factors that are associated with obesity. In addition, data based on self-reported dietary intake are prone to errors [57] including recall bias and social desirability bias, such that if self-reported dietary intake of nuts is recalled and reported differently than other foods, consumers of walnuts, other nuts, and nonconsumers may be misclassified [58]. Further, the participants who ate nuts but did not consume nuts on the day of data collection may have been classified as NN consumers.

In conclusion, despite being energy dense, consumption of tree nuts, specifically WON, was associated with a lower prevalence of obesity and a lower RFM compared to no consumption of nuts, especially in young women and girls. Because the findings of our current study are observational, future randomized controlled trials should determine the effect of consuming a combination of walnuts and other nuts for weight management in adolescents and young adults.

## Author contributions

The authors’ responsibilities were as follows – KT, NG-M: project conception, research design, manuscript writing; CDT: supervised data analysis, manuscript revision; BH: data analysis, manuscript writing/revision; and all authors: read and approved the final manuscript.

## Conflict of interest

NG-M has received funding from the Hass Avocado Board and New Capstone, and she has served on the Scientific Advisory Board of Haleon. BH has been involved in research for which her institution, Indiana University, has received grants from NIH, WW International, Inc., the Alliance of Potato Research & Education, the National Pork Board, and the National Cattlemen’s Beef Association. CDT was supported by the National Cancer Institute Supplemental Award U01-CA057030-29S2. All other authors report no conflicts of interest.

## Funding

This study was funded by the California Walnut Commission.

## Data availability

Data described in the manuscript, code book, and analytic code will be made available upon request pending application and approval. This study used publicly available data.
